# Data-driving methods: More than merely trendy buzzwords?

**DOI:** 10.1186/s13613-018-0405-7

**Published:** 2018-05-02

**Authors:** Julien Textoris, Fabio Silvio Taccone, Lara Zafrani, Antoine Guillon, Sébastien Gibot, Fabrice Uhel, Eric Azabou, Guillaume Monneret, Frédéric Pène, Nicolas de Prost, Stein Silva, the Translational Research Committee of the French Intensive Care Society (Société de Réanimation de Langue Française, SRLF)

**Affiliations:** 1 Département d’Anesthésie-Réanimation, hôpital Édouard-Herriot, Hospices Civils de Lyon, CHU de Lyon, 69437 Lyon, France; 2 Service de Soins Intensifs, Hôpital Erasme, 1070 Brussels, Belgium; 3 Service de Réanimation Médicale, APHP Hôpital Saint-Louis, Paris, France; 4 Service de Médecine Intensive - Réanimation, CHU de Tours, 37000 Tours, France; 5 Service de Réanimation Médicale, Hôpital Central, CHU de Nancy, 54000 Nancy, France; 6 Service de Réanimation Médicale et Maladies Infectieuses, Hôpital Pontchaillou, CHU de Rennes, Rennes, France; 7 Service de Réanimation, APHP Hôpital Raymond Poincaré, Garches, 92380 Paris, France; 8 Laboratoire d’immunologie, hôpital Edouard Herriot, Hospices Civils de Lyon, CHU de Lyon, 69437 Lyon, France; 9 Service de Réanimation Médicale, APHP, Hôpital Cochin, Paris, France; 10 Service de Réanimation Médicale, Hôpital Henri Mondor, 51, Avenue du Maréchal de Lattre de Tassigny, 94010 Créteil Cedex, France; 11 Service de Réanimation, CHU Purpan, 31300 Toulouse, France

**Keywords:** Big data, Machine learning, Artificial intelligence, Critical care

Intensive care units (ICU) physicians are experiencing a rapidly expanding collection of vast amounts of data from routine practice, patients’ monitoring as well as from diagnostic or prognostic tests. However, although these data could influence their clinical decisions and management, the validity and relevance of data processing methods, in particular in case of complex data sets (i.e. so-called big data, see Table 1 for related terminology) remain to be defined. A growing body of research has recently suggested that emerging artificial intelligence (AI)-derived methods could help physicians to access, organize and use important amounts of data more easily. Nowadays, such methods have already found applications in various fields, including technology, biology, computer science or sociology [1]. However, are these approaches more than merely trendy buzzwords? Are they reliable enough to match the exponential growth of medical complexity in the critical care setting? And, last but not least, can the holistic use of massive data sources available eventually provide clinically relevant information?

**Table 1:** Data-driven analysis and related terminology

Big data	Data sets with size/complexity beyond the capacity of commonly used methodological approaches to capture, manage and process data. Big data might be defined by their high *volume*, large *variety* and the important *velocity* that is required to process (3v definition)
Closed-loop system	System in which some or all its outputs are used as inputs. In health care, the use of such feedback loop enables real-time analysis of patient databases and could permit to optimize clinical care leading to more efficient targeting of tests and treatments and vigilance for adverse effects (i.e. dynamic clinical data mining)
Cross-validation	Statistical technique for assessing how the results of an analysis will generalize to an independent data set. For example, doing so it could permit to estimate how accurately a predictive model will perform in practice
Crowdsourcing	The practice of obtaining needed solution by soliciting contribution from a large group of people and specially from online communities
Data mining	The process of collecting, searching through and analysing a large amount of data in a database, as to discover patterns of relationships. It is worth noting that this approach does not look for causality and simply aim to detect significant data configurations
Machine learning	Derived methods from artificial intelligence that provides computers with the ability to learn without being explicitly programmed. The process of machine learning uses the data to detect patterns and adjust programme actions accordingly

The reality is that the exponential combinations of patients, conditions and treatments cannot be exhaustively explored by processes that often—intentionally or inadvertently—exclude interdependent input/output parameters because they do not fit into a priori hypotheses or predefined models (Additional file 1: Figure S1). In such a context, data-driven approaches hold promise of accurately dealing with big data methodological issues, and doing so might have a significant impact on the improvement in diagnosis, monitoring and prognostication of ICU processes.

## ICU database: closing the data loop

As an evolution to this approach, a dynamic clinical “data mining” (Table 1) has been recently proposed, based on “data-driven” methods (Additional file 1: Figure S1). The main idea is the use of feedback loops to enable real-time analysis of patient databases, allow the optimization of patient’s care and lead to more efficient targeting of tests, treatments and vigilance for adverse effects (e.g. “Multiparameter Intelligent Monitoring in Intensive Care” (MIMIC) [2]. Such closed-loop databases provide physicians with a unique opportunity to accumulate useful clinical evidence to: (1) identify patient subpopulations with important variations in treatment efficacy or unexpected delayed adverse effects, (2) reveal interactions between simultaneous treatments and physiological conditions, (3) create and cross-validate (Table 1) predictive models across research teams and institutions to better determine which findings are generalizable and (4) pave the way for the development and validation of innovative and more personalized treatments.

## Establishing knowledge

Big data methods seem to have straightforward applications for personalized medicine [3] and might pave the way for promising studies focused on the analysis of the intrinsic complexity underpinning human physiology.

### Omics: the rise of the narciss-ome

**Fig. 1:**
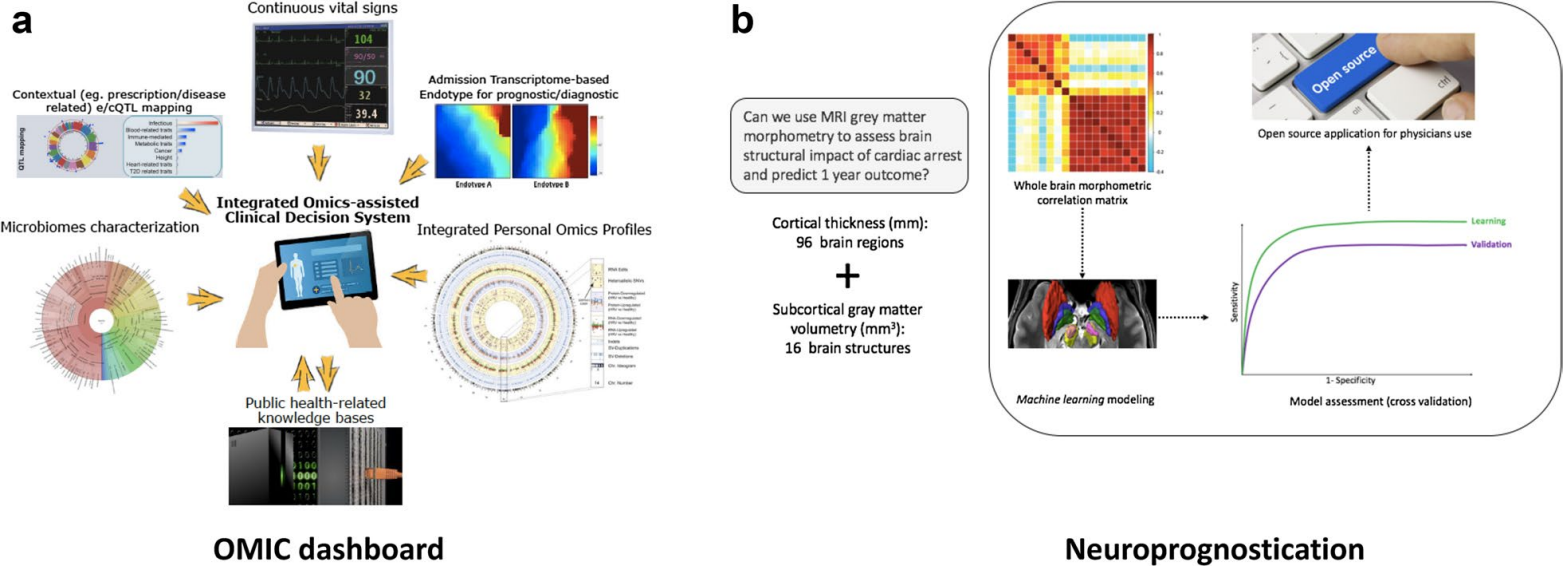
Data-driven methods applications*. A.* Potential ICU dashboard. It will integrate multimodal sources of big data, leveraging on continuous monitoring information, personal omics data sets, public health-related databases, medical notes and prescribed treatments. Probably, future ICU physicians will have to confront their medical assessment to integrated omics-assisted clinical decision systems, to ultimately provide more efficient, individual-tailored and real-time patient care. *B.* Neuroprognostication for cardiac arrest survivors. Use of early brain MRI grey matter morphometric data-driven analysis, to assess one-year neurological outcome after cardiac arrest

Omics data represent a massive source of multimodal data. The European Bioinformatics Institute (EBI), one of the world’s largest biological data repositories, is currently storing: ~ 5 petabytes (Additional file 2: Table S1) nucleotide sequence data, more than 30,000 genomes and ~ 2 million gene expression assays [3]. Furthermore, this infrastructure has been accessed 562 million times each month by ~ 9 million distinct hosts in 2015. These impressive figures highlight the fact that data-driven analysis methods are already a constituent part of worldwide collaborative research projects, built on large big data sharing (Fig. 1a).

### Brain, consciousness and complexity

To illustrate this point and demonstrate how data-driven approaches could be successfully used in this setting, we can describe a recent study focused on the assessment of brain structural impact of anoxic/hypoxic insult related to cardiac arrest (CA), and the potential use of brain MRI grey matter morphometry to predict patients’ one-year neurological outcome. The authors [4] studied a large and multicenter cohort of anoxic comatose patients, which were scanned during the acute phase following CA in standardized conditions. Crucially, to accurately evaluate whole-brain grey matter morphometry in this setting, fine-grained quantification techniques were applied. Eventually, a data-driven approach was used and permitted to obtain a predictive classifier that showed a significant discriminative power [4] and enabled the identification of brain grey structures whose degree of atrophy was significantly related to one-year neurological outcome (Fig. 1b).

### Promises, pitfalls and challenges

Complex statistical analyses designed to deal with large data sets might appear as magic bullets rendering cumbersome randomized trials dispensable (Additional file 3: Table S2). In fact, we should certainly keep in mind that these statistical optimization techniques are not shortcuts to broader medical reasoning and should not deter clinicians from carefully scrutinizing data so that to avoid inappropriate and naive use of these elegant analytical methods. For example, population selection and adjustment processes may dramatically influence the outcome of studies, giving rise to diametrically opposite conclusions [5].

Furthermore, few additional and unavoidable challenges, which are specifically related to the use of data-driven methods should be addressed: (1) computational issues should be adequately addressed probably by means of cloud storage and cloud computing facilities [6], (2) improving quality and ability to structure data, to ensure interoperability between various sources of data [7], (3) cultural and ethical issues should also be considered and constitute a still moot issue in the field, raising questions on data ownership, patient anonymity, agreement to participate and accountability [8], and highlight the need for further debate, standardization and update of the current legal or regulatory frameworks [9] and (4) finally, it is worth noting that the need for specific analytical skills (inference, prediction and computational abilities) justifies new collaborative interactions between research teams as well as specific training for both data scientist and future physicians [10].

## Conclusion

Considering the complexity of ICU setting, we have illustrated how data-driven approaches, through closed-loop systems integrating multimodal data, hold the promise to provide individually tailored and real-time patient care based on the large amount of information currently at our disposal. Regarding translational research, data-driven and hypothesis-driven approaches appear not to be mutually exclusive, but largely complementary and reciprocally challenging. Understanding the opportunities and pitfalls of implementing big data in the ICU setting and considering the subsequent technical, ethical and societal changes are key issues for the upcoming years, paving the way for critical diagnostic and therapeutic innovations.

## Additional files

**Additional file 1: Figure S1.** Analytical methods for biomedical research. Compared to rational hypothesis-driven research methods (upper panel), data-driven analysis (lower panel) does not imply reductions neither of the number of hypothesis that could be studied (i.e. including dynamical interactions), nor of the obtained data that is used to extract relevant information. Additionally, hypothesis-driven methods are built on optimised models derived from artificial intelligence domains, which can learn and evolve without explicit programming, and validate the created model using data from multiple and independent data sets (i.e. machine learning, supplementary-table-1 for related terminology).

**Additional file 2: Table S1** Conventional terms used to describe data size. Scale is based on powers of 1000.

**Additional file 3: Table S2** Opportunities and difficulties related to data-driven analysis.

## Abbreviations

AI: artificial intelligence
CA: cardiac arrest
EBI: European Bioinformatics Institute
ICU: intensive care unit
MIMIC: Multiparameter Intelligent Monitoring in Intensive Care
