# Supplementary figures and images for: Data-driving methods: More than merely trendy buzzwords?
Source: Ann Intensive Care. 2018 May 2;8:58. doi: 10.1186/s13613-018-0405-7 (PMC5931952; doi:10.1186/s13613-018-0405-7)

## Hypothesis-driven

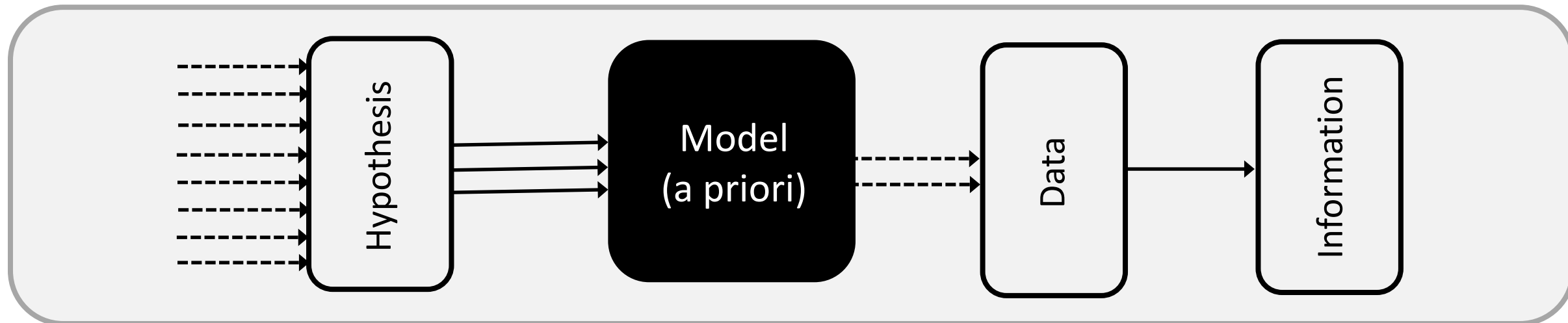

## Data-driven

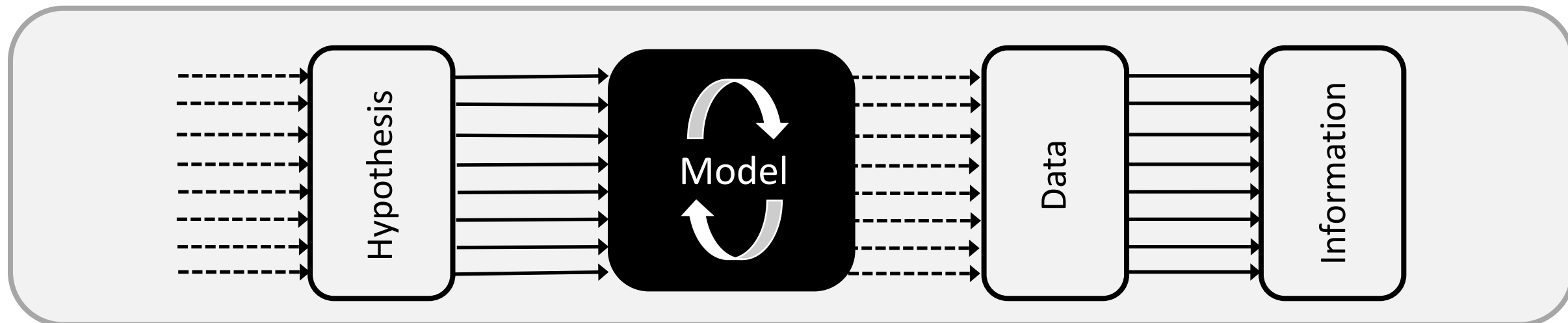

Supplement: Supplementary file 1 — Additional file 1: Figure S1. Analytical methods for biomedical research. Compared to rational hypothesis-driven research methods (upper panel), data-driven analysis (lower panel) does not imply reductions neither of the number of hypothesis that could be studied (i.e. including dynamical interactions), nor of the obtained data that is used to extract relevant information. Additionally, hypothesis-driven methods are built on optimised models derived from artificial intelligence domains, which can learn and evolve without explicit programming, and validate the created model using data from multiple and independent data sets (i.e. machine learning, supplementary-table-1 for related terminology). [file 13613_2018_405_MOESM1_ESM.pdf]
