# Supplementary material for: Data-driving methods: More than merely trendy buzzwords?
Source: Ann Intensive Care. 2018 May 2;8:58. doi: 10.1186/s13613-018-0405-7 (PMC5931952; doi:10.1186/s13613-018-0405-7)
Supplement: Supplementary file 2 — Additional file 2: Table S1 Conventional terms used to describe data size. Scale is based on powers of 1000. [file 13613_2018_405_MOESM2_ESM.doc]

| **Value** | **Metrics** | **Abbreviation** |
| --- | --- | --- |
| **1000** | Kilobyte | kB |
| **1000²** | Megabyte | MB |
| **1000^3^** | Gigabyte | GB |
| **1000^4^** | Terabyte | TB |
| **1000^5^** | Petabyte | PB |
| **1000^6^** | Exabyte | EB |
| **1000^7^** | Zettabyte | ZB |
| **1000^8^** | Yottabyte | YB |
