# Supplementary material for: Data-driving methods: More than merely trendy buzzwords?
Source: Ann Intensive Care. 2018 May 2;8:58. doi: 10.1186/s13613-018-0405-7 (PMC5931952; doi:10.1186/s13613-018-0405-7)
Supplement: Supplementary file 3 — Additional file 3: Table S2 Opportunities and difficulties related to data-driven analysis. [file 13613_2018_405_MOESM3_ESM.docx]

| Opportunities | Difficulties |
| --- | --- |
| Improve physiology understanding.  Capture systems interactions and adequately deal with multidimensional data complexity. | **Cost.**  Significant computational resources are needed in most of the cases. |
| Integrate clinical practice variability.  Allow “real-word” clinical studies, who appropriately consider clinical practice heterogeneity. | **Ethics.**  Ethical and cultural specific issues: data ownership, patient’s anonymity, difference between relationship and causality. |
| Closed loop system for healthcare.  Real-time analysis of patient databases, could lead to more efficient targeting of tests and treatments and vigilance for adverse effects. Additionally, it could be expected that these tools, permit to create and validated useful and accurate clinical practice guidelines and decision support systems. | **Naïve use.**  Artificial intelligence (AI) should aim to assist and not replace medical reasoning. There is a real risk of blind application of black-box statistical techniques for research. |
| Data sharing and collaborative research.  Outsourcing and crowdsourcing projects using worldwide network providing interdisciplinary ideas, cross-modal data, and updated methods for evidence creation. | **Specific skills.**  Role of “data scientists” owning interdisciplinary abilities and knowledge in diverse fields as mathematics, informatics, data monitoring and data management. |
| Teaching and learning.  Elaboration of dynamical learning system innovative online learning pathways. Artificial intelligence (AI) could assist healthcare learners with self-direction, self-assessment, interconnectedness and teamwork. |  |
